# Carbonyl­bis­(triphenyl­phosphane-κ*P*)(η^2^-1-vinyl­pyrrolidin-2-one-κ*O*)ruthenium(0)

**DOI:** 10.1107/S1600536812014766

**Published:** 2012-04-18

**Authors:** Si Jia Ma, Po Niu

**Affiliations:** a Department of Preventive Medicine, School of Public Health, Xiamen University, Xiamen 361005, Fujian, People’s Republic of China; b Department of Chemistry, College of Chemistry and Chemical Engineering, Xiamen University, Xiamen 361005, Fujian, People’s Republic of China

## Abstract

The 1-vinyl­pyrrolidin-2-one ligand in the title compound, [Ru(C_6_H_9_NO)(C_18_H_15_P)_2_(CO)], coordinates to the Ru^0^ atom with the olefin double bond and the ketone O atom. The Ru^0^ atom adopts a distorted trigonal–bipyramidal coordination geometry, with the C O ligand and the ketone O atom occupying the axial positions. The two triphenyl­phosphane ligands are *cis* to each other. The olefinic C=C bond is almost coplanar with the Ru^0^ atom and the two P atoms (maximum deviation of 0.0516 Å from the mean plane defined by the five constituent atoms). The coordinated C=C bond has a length of 1.449 (3) Å, which is significantly longer than that of a free C=C bond (1.34 Å). There are two C—H⋯π inter­actions involving neighbouring phenyl rings in the mol­ecule. In the crystal, mol­ecules are linked *via* two further C—H⋯π inter­actions.

## Related literature

For general background to ruthenium(0)-catalysed C—H activation, see: Murai *et al.* (1993 ▶). For C=C bond lengths for free olefinic double bonds, see: Orpen *et al.* (1989 ▶). For structurally related compounds, see: Lu *et al.* (1998 ▶); Jazzar *et al.* (2001 ▶).
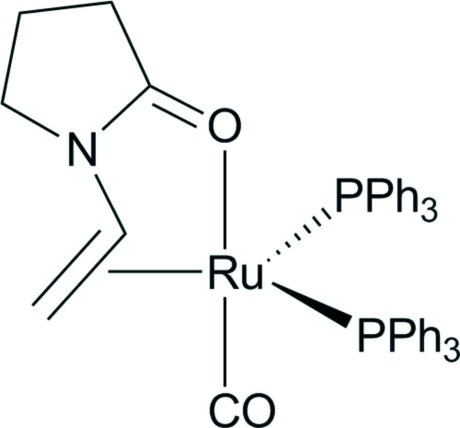


## Experimental

### 

#### Crystal data

[Ru(C_6_H_9_NO)(C_18_H_15_P)_2_(CO)]
*M* *_r_* = 764.76
Triclinic, 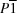

*a* = 10.765 (2) Å
*b* = 12.577 (3) Å
*c* = 13.878 (3) Å
α = 76.91 (3)°
β = 88.43 (3)°
γ = 83.89 (3)°
*V* = 1819.7 (6) Å^3^
*Z* = 2
Mo *K*α radiation
μ = 0.56 mm^−1^
*T* = 173 K
0.30 × 0.30 × 0.20 mm

#### Data collection

Rigaku R-AXIS RAPID IP diffractometer
Absorption correction: multi-scan (*ABSCOR*; Higashi, 1995 ▶) *T*_min_ = 0.753, *T*_max_ = 1.000
15733 measured reflections
7113 independent reflections
6570 reflections with *I* > 2σ(*I*)
*R*_int_ = 0.025

#### Refinement

*R*[*F* ^2^ > 2σ(*F* ^2^)] = 0.025
*wR*(*F* ^2^) = 0.084
*S* = 1.15
7113 reflections
442 parameters
H-atom parameters constrained
Δρ_max_ = 0.66 e Å^−3^
Δρ_min_ = −0.80 e Å^−3^

### 

Data collection: *CrystalClear* (Rigaku, 2000 ▶); cell refinement: *CrystalClear*; data reduction: *CrystalClear*; program(s) used to solve structure: *SHELXS97* (Sheldrick, 2008 ▶); program(s) used to refine structure: *SHELXL97* (Sheldrick, 2008 ▶); molecular graphics: *SHELXTL* (Sheldrick, 2008 ▶); software used to prepare material for publication: *SHELXTL*.

## Supplementary Material

Crystal structure: contains datablock(s) I, global. DOI: 10.1107/S1600536812014766/su2399sup1.cif

Structure factors: contains datablock(s) I. DOI: 10.1107/S1600536812014766/su2399Isup2.hkl

Additional supplementary materials:  crystallographic information; 3D view; checkCIF report

## Figures and Tables

**Table 1:** Hydrogen-bond geometry (Å, °) *Cg*1, *Cg*2 and *Cg*3 are the centroids of the C21–C26, C41–C46 and C61–C66 rings, respectively.

*D*—H⋯*A*	*D*—H	H⋯*A*	*D*⋯*A*	*D*—H⋯*A*
C36—H36*A*⋯*Cg*3	0.95	2.68	3.490 (2)	144
C66—H66*A*⋯*Cg*1	0.95	2.61	3.384 (3)	139
C4—H4*B*⋯*Cg*2^i^	0.99	2.67	3.585 (3)	154
C14—H14*A*⋯*Cg*3^ii^	0.95	2.90	3.693 (3)	142

Symmetry codes: (i) 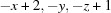; (ii) 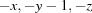.

**Fig. 1.**
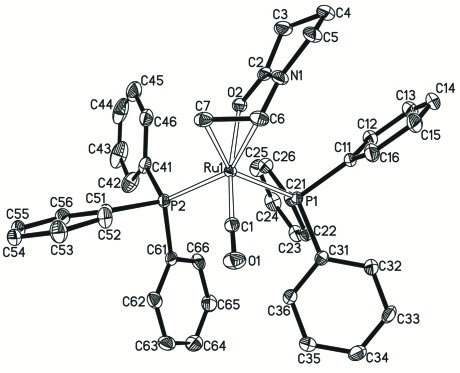
The molecular structure of the title compound with the atom-labelling. The displacement ellipsoids are drawn at the 40% probability level displacement ellipsoids (H atoms have been omitted for clarity).

## supplementary crystallographic information

### Comment

*Ortho*-alkylation of acetophenone with vinyl silanes *via* ruthenium catalyzed C—H activation has been reported by (Murai *et al.*, 1993) Cylometallation of the aromatic ketone with the catalytically active ruthenium(0) species, Ru(CO)(PPh_3_)_3_, generated from dehydrogenation of RuH_2_(CO)(PPh_3_)_3_, is proposed as the key step (Murai *et al.*, 1993). On exploring the feasibility of cyclometallation of *N*-vinyl-2-pyrrolidone with the ruthenium hydride complex RuH_2_(CO)(PPh_3_)_3_, the title compound was obtained instead of the cyclometallated product.

The molecular structure of the title compound is illustrated in Fig. 1. The *N*-Vinyl-2-pyrrolidone ligand is bound to the ruthenium(0) center via the olefin double bond (C6═C7) and the ketone O atom (O2). The ruthenium(0) atom, Ru1, adopts a distorted trigonal bipyramidal coordination geometry with the carbonyl ligand (C1═O1) and the ketone O atom, O2, occupying the axial positions. The two triphenylphosphane ligands are *cis* to each other. The olefin C6═C7 double bond is almost coplanar with atom Ru1 and the two P atoms (P1 and P2), as reflected by the small mean deviation of 0.0516 Å from the mean plane defined by the five constituent atoms.

The Ru1—C6 and Ru1—C7 bond distances (2.127 (2) and 2.157 (2) Å, respectively) are similar to those reported for related olefin coordinated ruthenium complexes, such as Ru(η^2^-*o*-acetylstyrene-O)(CO) (PPh_3_)_2_ [2.121 (8) and 2.167 (9) Å; Lu *et al.*, 1998], and Ru(PPh_3_)_3_(CO)(C_2_H_4_) [2.199 (8) and 2.213 (10) Å; Jazzar *et al.*, 2001]. The C6—C7 bond length of 1.449 (3) Å is significantly longer than that for a free olefinic double bond [1.34 Å; Orpen *et al.*, 1989], but is typical for a coordinated C═C double bond, for example as in Ru(η^2^-*o*-acetylstyrene-O)(CO)(PPh_3_)_2_ [1.43 (1) Å; Lu *et al.* 1998] and Ru(PPh_3_)_3_(CO)(C_2_H_4_) [1.451 (11) Å; Jazzar *et al.* 2001].

There are two C-H···π interactions involving neighbouring phenyl rings in the molecule, and in the crystal, molecules are linked via two further C-H···π interactions (Table 1).

### Experimental

To a solution of RuH_2_(CO)(PPh_3_)_3_ (0.40 g, 0.44 mmol) in toluene (20 ml) and under a nitrogen atmosphere was added *N*-vinyl-2-pyrrolidone (0.40 ml, 3.6 mmol). The reaction mixture was then refluxed for 1 h to give a yellow solution. After filtration, the filtrate was concentrated to *ca*. 1 ml under reduced pressure. 20 ml n-hexane were added to the residue with stirring to give a yellow solid. The solid was collected by filtration, washed with n-hexane and diethyl ether, and dried under vacuum [Yield: 0.24 g, 72%]. Yellow block-like crystals, suitable for X-ray analysis, were obtained by layering a dichloromethane solution of the title compound with hexane.

### Refinement

The H atoms were included in calculated positions and treated as rding atoms: C—H = 0.95, 0.99, 0.99 and 1.00 Å for phenyl, pyrrolidone, CH_2_ and CH H atoms, respectively, with U_iso_(H) = k × *U*_eq_(C), where k = 1.5 for pyrrolidone H atoms, and = 1.2 for other H atoms. In the final difference Fourier map the highest and lowest residual electron density peaks were 0.94 and 0.92 Å, respectively, from atom Ru1.

### Crystal data

**Table:**

[Ru(C_6_H_9_NO)(C_18_H_15_P)_2_(CO)]	*Z* = 2
*M**_r_* = 764.76	*F*(000) = 788
Triclinic, *P*1	*D*_x_ = 1.396 Mg m^−^^3^
Hall symbol: -P 1	Mo *K*α radiation, λ = 0.71073 Å
*a* = 10.765 (2) Å	Cell parameters from 16802 reflections
*b* = 12.577 (3) Å	θ = 6.0–55.0°
*c* = 13.878 (3) Å	µ = 0.56 mm^−^^1^
α = 76.91 (3)°	*T* = 173 K
β = 88.43 (3)°	Block, yellow
γ = 83.89 (3)°	0.30 × 0.30 × 0.20 mm
*V* = 1819.7 (6) Å^3^	

### Data collection

**Table:**

Rigaku R-AXIS RAPID IP diffractometer	7113 independent reflections
Radiation source: fine-focus sealed tube	6570 reflections with *I* > 2σ(*I*)
Graphite monochromator	*R*_int_ = 0.025
Oscillation scans	θ_max_ = 26.0°, θ_min_ = 3.0°
Absorption correction: multi-scan (*ABSCOR*; Higashi, 1995)	*h* = −13→13
*T*_min_ = 0.753, *T*_max_ = 1.000	*k* = −14→15
15733 measured reflections	*l* = −16→17

### Refinement

**Table:**

Refinement on *F*^2^	Primary atom site location: structure-invariant direct methods
Least-squares matrix: full	Secondary atom site location: difference Fourier map
*R*[*F*^2^ > 2σ(*F*^2^)] = 0.025	Hydrogen site location: inferred from neighbouring sites
*wR*(*F*^2^) = 0.084	H-atom parameters constrained
*S* = 1.15	*w* = 1/[σ^2^(*F*_o_^2^) + (0.0493*P*)^2^ + 0.4957*P*] where *P* = (*F*_o_^2^ + 2*F*_c_^2^)/3
7113 reflections	(Δ/σ)_max_ = 0.002
442 parameters	Δρ_max_ = 0.66 e Å^−^^3^
0 restraints	Δρ_min_ = −0.80 e Å^−^^3^

### Special details

**Table:**

Geometry. All e.s.d.'s (except the e.s.d. in the dihedral angle between two l.s. planes) are estimated using the full covariance matrix. The cell e.s.d.'s are taken into account individually in the estimation of e.s.d.'s in distances, angles and torsion angles; correlations between e.s.d.'s in cell parameters are only used when they are defined by crystal symmetry. An approximate (isotropic) treatment of cell e.s.d.'s is used for estimating e.s.d.'s involving l.s. planes.
Refinement. Refinement of *F*^2^ against ALL reflections. The weighted *R*-factor *wR* and goodness of fit *S* are based on *F*^2^, conventional *R*-factors *R* are based on *F*, with *F* set to zero for negative *F*^2^. The threshold expression of *F*^2^ > σ(*F*^2^) is used only for calculating *R*-factors(gt) *etc*. and is not relevant to the choice of reflections for refinement. *R*-factors based on *F*^2^ are statistically about twice as large as those based on *F*, and *R*- factors based on ALL data will be even larger.

### Fractional atomic coordinates and isotropic or equivalent isotropic displacement parameters (Å^2^)

**Table:**

	*x*	*y*	*z*	*U*_iso_*/*U*_eq_	
Ru1	1.093351 (13)	0.114104 (11)	0.257193 (10)	0.01529 (7)	
P1	1.25572 (5)	0.07132 (4)	0.15043 (4)	0.01623 (11)	
P2	1.13168 (5)	0.28498 (4)	0.28814 (4)	0.01797 (11)	
C1	0.99017 (19)	0.17719 (16)	0.15533 (15)	0.0203 (4)	
O1	0.92111 (15)	0.21343 (14)	0.08981 (12)	0.0338 (4)	
O2	1.21345 (13)	0.00891 (11)	0.37587 (10)	0.0218 (3)	
N1	1.08507 (17)	−0.11518 (14)	0.35553 (13)	0.0254 (4)	
C2	1.1837 (2)	−0.08766 (17)	0.39478 (15)	0.0234 (4)	
C3	1.2510 (2)	−0.18642 (19)	0.46185 (18)	0.0340 (5)	
H3A	1.2313	−0.1881	0.5322	0.041*	
H3B	1.3425	−0.1886	0.4519	0.041*	
C4	1.1982 (3)	−0.28127 (19)	0.42819 (19)	0.0411 (6)	
H4A	1.2525	−0.3061	0.3771	0.049*	
H4B	1.1906	−0.3443	0.4848	0.049*	
C5	1.0699 (3)	−0.23248 (18)	0.38591 (19)	0.0359 (6)	
H5A	1.0498	−0.2624	0.3287	0.043*	
H5B	1.0036	−0.2466	0.4367	0.043*	
C6	0.9984 (2)	−0.02938 (17)	0.29936 (16)	0.0244 (4)	
H6A	0.9470	−0.0486	0.2480	0.029*	
C7	0.9423 (2)	0.05170 (18)	0.35122 (16)	0.0257 (4)	
H7A	0.9564	0.0361	0.4235	0.031*	
H7B	0.8568	0.0850	0.3311	0.031*	
C11	1.2866 (2)	−0.07769 (16)	0.15914 (15)	0.0207 (4)	
C12	1.4022 (2)	−0.13692 (18)	0.18325 (16)	0.0280 (5)	
H12A	1.4706	−0.1004	0.1962	0.034*	
C13	1.4186 (3)	−0.24953 (19)	0.18854 (18)	0.0377 (6)	
H13A	1.4981	−0.2894	0.2050	0.045*	
C14	1.3193 (3)	−0.30354 (19)	0.16991 (18)	0.0387 (6)	
H14A	1.3304	−0.3805	0.1744	0.046*	
C15	1.2040 (3)	−0.24510 (19)	0.14469 (18)	0.0357 (6)	
H15A	1.1362	−0.2818	0.1308	0.043*	
C16	1.1871 (2)	−0.13309 (18)	0.13966 (17)	0.0282 (5)	
H16A	1.1075	−0.0936	0.1229	0.034*	
C21	1.41278 (18)	0.10880 (15)	0.16887 (15)	0.0202 (4)	
C22	1.4889 (2)	0.15284 (17)	0.09011 (16)	0.0238 (4)	
H22A	1.4639	0.1574	0.0241	0.029*	
C23	1.6017 (2)	0.19015 (18)	0.10803 (18)	0.0289 (5)	
H23A	1.6526	0.2213	0.0542	0.035*	
C24	1.6393 (2)	0.1817 (2)	0.20374 (19)	0.0334 (5)	
H24A	1.7159	0.2076	0.2158	0.040*	
C25	1.5658 (2)	0.1357 (2)	0.28275 (18)	0.0330 (5)	
H25A	1.5931	0.1285	0.3487	0.040*	
C26	1.4523 (2)	0.10020 (18)	0.26531 (16)	0.0255 (4)	
H26A	1.4014	0.0699	0.3195	0.031*	
C31	1.23428 (18)	0.12472 (16)	0.01664 (14)	0.0194 (4)	
C32	1.2646 (2)	0.06049 (17)	−0.05196 (16)	0.0249 (4)	
H32A	1.2939	−0.0149	−0.0296	0.030*	
C33	1.2523 (2)	0.10559 (19)	−0.15253 (16)	0.0289 (5)	
H33A	1.2727	0.0609	−0.1986	0.035*	
C34	1.2106 (2)	0.2149 (2)	−0.18598 (16)	0.0313 (5)	
H34A	1.2024	0.2455	−0.2550	0.038*	
C35	1.1807 (2)	0.28009 (18)	−0.11915 (16)	0.0302 (5)	
H35A	1.1528	0.3556	−0.1423	0.036*	
C36	1.1914 (2)	0.23534 (17)	−0.01796 (15)	0.0239 (4)	
H36A	1.1695	0.2802	0.0277	0.029*	
C41	1.2382 (2)	0.28262 (18)	0.39059 (16)	0.0255 (4)	
C42	1.3214 (2)	0.3601 (2)	0.39016 (19)	0.0385 (6)	
H42A	1.3249	0.4201	0.3348	0.046*	
C43	1.3997 (3)	0.3501 (3)	0.4707 (2)	0.0536 (8)	
H43A	1.4570	0.4029	0.4695	0.064*	
C44	1.3949 (3)	0.2651 (3)	0.5516 (2)	0.0562 (9)	
H44A	1.4485	0.2589	0.6063	0.067*	
C45	1.3116 (3)	0.1881 (2)	0.5534 (2)	0.0517 (8)	
H45A	1.3077	0.1291	0.6095	0.062*	
C46	1.2340 (3)	0.1969 (2)	0.47356 (17)	0.0354 (5)	
H46A	1.1771	0.1437	0.4754	0.042*	
C51	0.9930 (2)	0.37120 (16)	0.31970 (15)	0.0214 (4)	
C52	0.8757 (2)	0.35118 (18)	0.29333 (17)	0.0289 (5)	
H52A	0.8681	0.2895	0.2659	0.035*	
C53	0.7688 (2)	0.4197 (2)	0.30621 (19)	0.0340 (5)	
H53A	0.6896	0.4058	0.2860	0.041*	
C54	0.7788 (2)	0.50788 (18)	0.34849 (16)	0.0304 (5)	
H54A	0.7064	0.5548	0.3576	0.037*	
C55	0.8944 (2)	0.52760 (18)	0.37747 (17)	0.0309 (5)	
H55A	0.9009	0.5875	0.4075	0.037*	
C56	1.0012 (2)	0.46060 (17)	0.36303 (16)	0.0270 (5)	
H56A	1.0803	0.4755	0.3826	0.032*	
C61	1.1924 (2)	0.38446 (15)	0.18363 (14)	0.0203 (4)	
C62	1.1122 (2)	0.46428 (17)	0.12252 (17)	0.0266 (5)	
H62A	1.0270	0.4754	0.1408	0.032*	
C63	1.1553 (2)	0.52794 (18)	0.03498 (17)	0.0317 (5)	
H63A	1.0993	0.5819	−0.0060	0.038*	
C64	1.2787 (3)	0.51297 (18)	0.00759 (18)	0.0346 (6)	
H64A	1.3078	0.5561	−0.0524	0.042*	
C65	1.3605 (2)	0.43484 (18)	0.06782 (17)	0.0313 (5)	
H65A	1.4458	0.4249	0.0492	0.038*	
C66	1.3180 (2)	0.37086 (17)	0.15569 (17)	0.0257 (4)	
H66A	1.3746	0.3178	0.1968	0.031*	

### Atomic displacement parameters (Å^2^)

**Table:**

	*U*^11^	*U*^22^	*U*^33^	*U*^12^	*U*^13^	*U*^23^
Ru1	0.01573 (10)	0.01426 (10)	0.01555 (10)	−0.00144 (6)	0.00113 (6)	−0.00286 (6)
P1	0.0171 (3)	0.0156 (2)	0.0165 (2)	−0.00204 (19)	0.00124 (18)	−0.00476 (18)
P2	0.0192 (3)	0.0173 (2)	0.0182 (2)	−0.00184 (19)	0.00067 (19)	−0.00559 (19)
C1	0.0208 (10)	0.0194 (10)	0.0207 (10)	−0.0041 (8)	0.0045 (8)	−0.0042 (8)
O1	0.0273 (9)	0.0407 (9)	0.0286 (9)	0.0019 (7)	−0.0075 (7)	0.0005 (7)
O2	0.0219 (8)	0.0212 (7)	0.0209 (7)	−0.0007 (6)	0.0007 (6)	−0.0028 (6)
N1	0.0294 (10)	0.0167 (8)	0.0279 (10)	−0.0049 (7)	0.0051 (8)	0.0000 (7)
C2	0.0249 (11)	0.0238 (11)	0.0182 (10)	0.0034 (8)	0.0075 (8)	−0.0014 (8)
C3	0.0386 (14)	0.0280 (12)	0.0285 (12)	0.0070 (10)	0.0027 (10)	0.0031 (9)
C4	0.0614 (18)	0.0222 (12)	0.0332 (13)	0.0057 (11)	0.0091 (12)	0.0020 (9)
C5	0.0495 (16)	0.0192 (11)	0.0362 (13)	−0.0083 (10)	0.0134 (11)	0.0003 (9)
C6	0.0218 (11)	0.0217 (10)	0.0277 (11)	−0.0074 (8)	0.0005 (8)	0.0006 (8)
C7	0.0204 (11)	0.0281 (11)	0.0239 (11)	−0.0009 (8)	0.0050 (8)	0.0024 (8)
C11	0.0276 (11)	0.0158 (9)	0.0191 (10)	−0.0021 (8)	0.0039 (8)	−0.0051 (7)
C12	0.0317 (12)	0.0238 (11)	0.0285 (11)	0.0016 (9)	−0.0012 (9)	−0.0075 (9)
C13	0.0511 (16)	0.0242 (12)	0.0348 (13)	0.0109 (11)	−0.0053 (11)	−0.0068 (10)
C14	0.0672 (19)	0.0179 (11)	0.0310 (13)	−0.0007 (11)	0.0009 (12)	−0.0077 (9)
C15	0.0520 (16)	0.0239 (12)	0.0363 (13)	−0.0141 (11)	0.0054 (11)	−0.0134 (10)
C16	0.0313 (12)	0.0238 (11)	0.0316 (12)	−0.0037 (9)	0.0023 (9)	−0.0106 (9)
C21	0.0163 (10)	0.0156 (9)	0.0298 (11)	−0.0004 (7)	0.0019 (8)	−0.0079 (8)
C22	0.0226 (11)	0.0235 (10)	0.0260 (11)	−0.0016 (8)	0.0046 (8)	−0.0082 (8)
C23	0.0228 (11)	0.0257 (11)	0.0390 (13)	−0.0062 (9)	0.0098 (9)	−0.0086 (9)
C24	0.0201 (11)	0.0335 (12)	0.0498 (15)	−0.0077 (9)	−0.0007 (10)	−0.0134 (11)
C25	0.0272 (12)	0.0412 (13)	0.0328 (12)	−0.0040 (10)	−0.0073 (9)	−0.0119 (10)
C26	0.0231 (11)	0.0276 (11)	0.0263 (11)	−0.0041 (9)	0.0008 (8)	−0.0061 (9)
C31	0.0182 (10)	0.0222 (10)	0.0185 (10)	−0.0034 (8)	0.0011 (7)	−0.0057 (8)
C32	0.0273 (11)	0.0234 (10)	0.0244 (11)	0.0006 (9)	0.0000 (8)	−0.0076 (8)
C33	0.0349 (13)	0.0356 (12)	0.0186 (10)	−0.0004 (10)	0.0015 (9)	−0.0125 (9)
C34	0.0364 (13)	0.0357 (12)	0.0201 (11)	−0.0017 (10)	−0.0015 (9)	−0.0035 (9)
C35	0.0387 (14)	0.0233 (11)	0.0255 (11)	0.0009 (9)	0.0003 (9)	−0.0010 (9)
C36	0.0293 (12)	0.0227 (10)	0.0201 (10)	−0.0028 (9)	0.0030 (8)	−0.0062 (8)
C41	0.0236 (11)	0.0332 (11)	0.0217 (10)	0.0034 (9)	−0.0004 (8)	−0.0133 (9)
C42	0.0341 (14)	0.0547 (16)	0.0339 (13)	−0.0127 (12)	0.0008 (10)	−0.0214 (12)
C43	0.0308 (15)	0.091 (2)	0.0545 (19)	−0.0096 (15)	−0.0020 (13)	−0.0469 (18)
C44	0.0420 (17)	0.095 (2)	0.0378 (16)	0.0257 (16)	−0.0180 (13)	−0.0404 (17)
C45	0.072 (2)	0.0553 (17)	0.0258 (13)	0.0253 (15)	−0.0134 (13)	−0.0173 (12)
C46	0.0477 (15)	0.0344 (12)	0.0244 (12)	0.0089 (11)	−0.0024 (10)	−0.0131 (10)
C51	0.0247 (11)	0.0187 (10)	0.0205 (10)	0.0000 (8)	0.0034 (8)	−0.0053 (8)
C52	0.0259 (12)	0.0287 (11)	0.0356 (12)	0.0001 (9)	0.0007 (9)	−0.0161 (9)
C53	0.0250 (12)	0.0362 (13)	0.0420 (14)	0.0026 (10)	0.0012 (10)	−0.0141 (11)
C54	0.0353 (13)	0.0252 (11)	0.0266 (11)	0.0078 (9)	0.0087 (9)	−0.0030 (9)
C55	0.0446 (14)	0.0199 (10)	0.0292 (12)	−0.0016 (10)	0.0092 (10)	−0.0092 (9)
C56	0.0332 (13)	0.0219 (10)	0.0276 (11)	−0.0047 (9)	0.0028 (9)	−0.0083 (9)
C61	0.0281 (11)	0.0163 (9)	0.0186 (10)	−0.0050 (8)	0.0015 (8)	−0.0073 (7)
C62	0.0296 (12)	0.0208 (10)	0.0304 (12)	−0.0023 (9)	−0.0028 (9)	−0.0079 (9)
C63	0.0460 (15)	0.0203 (10)	0.0280 (12)	−0.0054 (10)	−0.0069 (10)	−0.0020 (9)
C64	0.0521 (16)	0.0235 (11)	0.0295 (12)	−0.0174 (11)	0.0070 (10)	−0.0032 (9)
C65	0.0354 (13)	0.0257 (11)	0.0347 (12)	−0.0119 (10)	0.0121 (10)	−0.0079 (9)
C66	0.0277 (11)	0.0170 (10)	0.0341 (12)	−0.0043 (8)	0.0032 (9)	−0.0085 (8)

### Geometric parameters (Å, º)

**Table:**

Ru1—P1	2.3578 (9)	C24—H24A	0.9500
Ru1—P2	2.3643 (7)	C25—C26	1.391 (3)
Ru1—O2	2.2135 (17)	C25—H25A	0.9500
Ru1—C1	1.803 (2)	C26—H26A	0.9500
Ru1—C6	2.127 (2)	C31—C32	1.393 (3)
Ru1—C7	2.157 (2)	C31—C36	1.397 (3)
P1—C31	1.838 (2)	C32—C33	1.387 (3)
P1—C21	1.842 (2)	C32—H32A	0.9500
P1—C11	1.845 (2)	C33—C34	1.378 (3)
P2—C61	1.843 (2)	C33—H33A	0.9500
P2—C41	1.844 (2)	C34—C35	1.381 (3)
P2—C51	1.852 (2)	C34—H34A	0.9500
C1—O1	1.168 (3)	C35—C36	1.393 (3)
O2—C2	1.257 (3)	C35—H35A	0.9500
N1—C2	1.318 (3)	C36—H36A	0.9500
N1—C6	1.445 (3)	C41—C46	1.391 (3)
N1—C5	1.465 (3)	C41—C42	1.392 (3)
C2—C3	1.502 (3)	C42—C43	1.393 (4)
C3—C4	1.541 (4)	C42—H42A	0.9500
C3—H3A	0.9900	C43—C44	1.368 (5)
C3—H3B	0.9900	C43—H43A	0.9500
C4—C5	1.526 (4)	C44—C45	1.384 (5)
C4—H4A	0.9900	C44—H44A	0.9500
C4—H4B	0.9900	C45—C46	1.383 (4)
C5—H5A	0.9900	C45—H45A	0.9500
C5—H5B	0.9900	C46—H46A	0.9500
C6—C7	1.449 (3)	C51—C52	1.388 (3)
C6—H6A	1.0000	C51—C56	1.401 (3)
C7—H7A	0.9900	C52—C53	1.395 (3)
C7—H7B	0.9900	C52—H52A	0.9500
C11—C12	1.388 (3)	C53—C54	1.382 (3)
C11—C16	1.402 (3)	C53—H53A	0.9500
C12—C13	1.394 (3)	C54—C55	1.383 (4)
C12—H12A	0.9500	C54—H54A	0.9500
C13—C14	1.385 (4)	C55—C56	1.389 (3)
C13—H13A	0.9500	C55—H55A	0.9500
C14—C15	1.385 (4)	C56—H56A	0.9500
C14—H14A	0.9500	C61—C62	1.393 (3)
C15—C16	1.387 (3)	C61—C66	1.400 (3)
C15—H15A	0.9500	C62—C63	1.393 (3)
C16—H16A	0.9500	C62—H62A	0.9500
C21—C26	1.393 (3)	C63—C64	1.377 (4)
C21—C22	1.395 (3)	C63—H63A	0.9500
C22—C23	1.396 (3)	C64—C65	1.387 (4)
C22—H22A	0.9500	C64—H64A	0.9500
C23—C24	1.377 (3)	C65—C66	1.396 (3)
C23—H23A	0.9500	C65—H65A	0.9500
C24—C25	1.387 (3)	C66—H66A	0.9500
			
C1—Ru1—C6	94.36 (9)	C21—C22—H22A	119.9
C1—Ru1—C7	92.91 (9)	C23—C22—H22A	119.9
C6—Ru1—C7	39.52 (9)	C24—C23—C22	120.0 (2)
C1—Ru1—O2	169.82 (7)	C24—C23—H23A	120.0
C6—Ru1—O2	77.10 (7)	C22—C23—H23A	120.0
C7—Ru1—O2	84.04 (7)	C23—C24—C25	120.4 (2)
C1—Ru1—P1	92.41 (7)	C23—C24—H24A	119.8
C6—Ru1—P1	105.69 (7)	C25—C24—H24A	119.8
C7—Ru1—P1	145.13 (6)	C24—C25—C26	119.9 (2)
O2—Ru1—P1	84.72 (5)	C24—C25—H25A	120.0
C1—Ru1—P2	92.95 (7)	C26—C25—H25A	120.0
C6—Ru1—P2	147.84 (6)	C25—C26—C21	120.3 (2)
C7—Ru1—P2	108.87 (7)	C25—C26—H26A	119.8
O2—Ru1—P2	97.22 (5)	C21—C26—H26A	119.8
P1—Ru1—P2	105.23 (3)	C32—C31—C36	118.67 (19)
C31—P1—C21	101.69 (10)	C32—C31—P1	122.55 (16)
C31—P1—C11	101.93 (9)	C36—C31—P1	118.72 (15)
C21—P1—C11	101.87 (10)	C33—C32—C31	120.6 (2)
C31—P1—Ru1	118.18 (7)	C33—C32—H32A	119.7
C21—P1—Ru1	118.42 (7)	C31—C32—H32A	119.7
C11—P1—Ru1	112.27 (7)	C34—C33—C32	120.3 (2)
C61—P2—C41	103.74 (10)	C34—C33—H33A	119.8
C61—P2—C51	99.15 (10)	C32—C33—H33A	119.8
C41—P2—C51	101.51 (10)	C33—C34—C35	119.9 (2)
C61—P2—Ru1	116.54 (7)	C33—C34—H34A	120.0
C41—P2—Ru1	117.30 (8)	C35—C34—H34A	120.0
C51—P2—Ru1	115.93 (7)	C34—C35—C36	120.2 (2)
O1—C1—Ru1	176.85 (17)	C34—C35—H35A	119.9
C2—O2—Ru1	110.30 (13)	C36—C35—H35A	119.9
C2—N1—C6	118.82 (17)	C35—C36—C31	120.3 (2)
C2—N1—C5	113.4 (2)	C35—C36—H36A	119.9
C6—N1—C5	127.36 (19)	C31—C36—H36A	119.9
O2—C2—N1	122.62 (19)	C46—C41—C42	118.4 (2)
O2—C2—C3	127.0 (2)	C46—C41—P2	117.04 (17)
N1—C2—C3	110.39 (19)	C42—C41—P2	124.53 (18)
C2—C3—C4	101.8 (2)	C41—C42—C43	120.2 (3)
C2—C3—H3A	111.4	C41—C42—H42A	119.9
C4—C3—H3A	111.4	C43—C42—H42A	119.9
C2—C3—H3B	111.4	C44—C43—C42	120.6 (3)
C4—C3—H3B	111.4	C44—C43—H43A	119.7
H3A—C3—H3B	109.3	C42—C43—H43A	119.7
C5—C4—C3	104.42 (19)	C43—C44—C45	119.7 (3)
C5—C4—H4A	110.9	C43—C44—H44A	120.1
C3—C4—H4A	110.9	C45—C44—H44A	120.1
C5—C4—H4B	110.9	C46—C45—C44	120.1 (3)
C3—C4—H4B	110.9	C46—C45—H45A	119.9
H4A—C4—H4B	108.9	C44—C45—H45A	119.9
N1—C5—C4	102.4 (2)	C45—C46—C41	120.9 (3)
N1—C5—H5A	111.3	C45—C46—H46A	119.6
C4—C5—H5A	111.3	C41—C46—H46A	119.6
N1—C5—H5B	111.3	C52—C51—C56	118.2 (2)
C4—C5—H5B	111.3	C52—C51—P2	118.60 (15)
H5A—C5—H5B	109.2	C56—C51—P2	123.10 (17)
N1—C6—C7	116.14 (19)	C51—C52—C53	121.4 (2)
N1—C6—Ru1	107.40 (13)	C51—C52—H52A	119.3
C7—C6—Ru1	71.35 (11)	C53—C52—H52A	119.3
N1—C6—H6A	117.6	C54—C53—C52	119.6 (2)
C7—C6—H6A	117.6	C54—C53—H53A	120.2
Ru1—C6—H6A	117.6	C52—C53—H53A	120.2
C6—C7—Ru1	69.13 (11)	C53—C54—C55	119.8 (2)
C6—C7—H7A	116.7	C53—C54—H54A	120.1
Ru1—C7—H7A	116.7	C55—C54—H54A	120.1
C6—C7—H7B	116.7	C54—C55—C56	120.6 (2)
Ru1—C7—H7B	116.7	C54—C55—H55A	119.7
H7A—C7—H7B	113.8	C56—C55—H55A	119.7
C12—C11—C16	118.81 (19)	C55—C56—C51	120.4 (2)
C12—C11—P1	123.66 (16)	C55—C56—H56A	119.8
C16—C11—P1	117.53 (17)	C51—C56—H56A	119.8
C11—C12—C13	120.5 (2)	C62—C61—C66	118.4 (2)
C11—C12—H12A	119.8	C62—C61—P2	121.09 (17)
C13—C12—H12A	119.8	C66—C61—P2	119.89 (16)
C14—C13—C12	120.2 (2)	C63—C62—C61	121.0 (2)
C14—C13—H13A	119.9	C63—C62—H62A	119.5
C12—C13—H13A	119.9	C61—C62—H62A	119.5
C15—C14—C13	119.8 (2)	C64—C63—C62	120.2 (2)
C15—C14—H14A	120.1	C64—C63—H63A	119.9
C13—C14—H14A	120.1	C62—C63—H63A	119.9
C14—C15—C16	120.2 (2)	C63—C64—C65	119.9 (2)
C14—C15—H15A	119.9	C63—C64—H64A	120.1
C16—C15—H15A	119.9	C65—C64—H64A	120.1
C15—C16—C11	120.5 (2)	C64—C65—C66	120.2 (2)
C15—C16—H16A	119.8	C64—C65—H65A	119.9
C11—C16—H16A	119.8	C66—C65—H65A	119.9
C26—C21—C22	119.16 (18)	C65—C66—C61	120.4 (2)
C26—C21—P1	118.23 (15)	C65—C66—H66A	119.8
C22—C21—P1	122.43 (16)	C61—C66—H66A	119.8
C21—C22—C23	120.2 (2)		
			
C1—Ru1—P1—C31	6.89 (9)	P1—C11—C12—C13	179.94 (18)
C6—Ru1—P1—C31	102.11 (10)	C11—C12—C13—C14	−0.1 (4)
C7—Ru1—P1—C31	105.47 (13)	C12—C13—C14—C15	0.8 (4)
O2—Ru1—P1—C31	177.11 (8)	C13—C14—C15—C16	−1.1 (4)
P2—Ru1—P1—C31	−86.83 (8)	C14—C15—C16—C11	0.6 (4)
C1—Ru1—P1—C21	130.35 (10)	C12—C11—C16—C15	0.2 (3)
C6—Ru1—P1—C21	−134.43 (10)	P1—C11—C16—C15	179.86 (17)
C7—Ru1—P1—C21	−131.07 (13)	C31—P1—C21—C26	167.81 (17)
O2—Ru1—P1—C21	−59.43 (9)	C11—P1—C21—C26	−87.17 (17)
P2—Ru1—P1—C21	36.63 (8)	Ru1—P1—C21—C26	36.48 (18)
C1—Ru1—P1—C11	−111.33 (10)	C31—P1—C21—C22	−7.30 (19)
C6—Ru1—P1—C11	−16.11 (10)	C11—P1—C21—C22	97.73 (18)
C7—Ru1—P1—C11	−12.75 (13)	Ru1—P1—C21—C22	−138.62 (15)
O2—Ru1—P1—C11	58.89 (9)	C26—C21—C22—C23	−1.5 (3)
P2—Ru1—P1—C11	154.95 (7)	P1—C21—C22—C23	173.53 (16)
C1—Ru1—P2—C61	−56.04 (10)	C21—C22—C23—C24	1.2 (3)
C6—Ru1—P2—C61	−159.05 (13)	C22—C23—C24—C25	0.4 (4)
C7—Ru1—P2—C61	−150.13 (10)	C23—C24—C25—C26	−1.5 (4)
O2—Ru1—P2—C61	123.73 (9)	C24—C25—C26—C21	1.1 (4)
P1—Ru1—P2—C61	37.27 (8)	C22—C21—C26—C25	0.4 (3)
C1—Ru1—P2—C41	−179.86 (10)	P1—C21—C26—C25	−174.87 (18)
C6—Ru1—P2—C41	77.12 (14)	C21—P1—C31—C32	93.05 (18)
C7—Ru1—P2—C41	86.05 (10)	C11—P1—C31—C32	−11.92 (19)
O2—Ru1—P2—C41	−0.09 (9)	Ru1—P1—C31—C32	−135.47 (16)
P1—Ru1—P2—C41	−86.56 (8)	C21—P1—C31—C36	−83.90 (17)
C1—Ru1—P2—C51	60.09 (10)	C11—P1—C31—C36	171.13 (16)
C6—Ru1—P2—C51	−42.92 (14)	Ru1—P1—C31—C36	47.57 (18)
C7—Ru1—P2—C51	−34.00 (10)	C36—C31—C32—C33	0.0 (3)
O2—Ru1—P2—C51	−120.14 (9)	P1—C31—C32—C33	−176.98 (17)
P1—Ru1—P2—C51	153.40 (7)	C31—C32—C33—C34	0.4 (3)
C6—Ru1—C1—O1	−12 (3)	C32—C33—C34—C35	−0.1 (4)
C7—Ru1—C1—O1	−52 (3)	C33—C34—C35—C36	−0.6 (4)
O2—Ru1—C1—O1	20 (4)	C34—C35—C36—C31	1.0 (3)
P1—Ru1—C1—O1	94 (3)	C32—C31—C36—C35	−0.7 (3)
P2—Ru1—C1—O1	−161 (3)	P1—C31—C36—C35	176.41 (17)
C1—Ru1—O2—C2	−20.1 (4)	C61—P2—C41—C46	−165.70 (17)
C6—Ru1—O2—C2	13.36 (14)	C51—P2—C41—C46	91.78 (19)
C7—Ru1—O2—C2	52.86 (14)	Ru1—P2—C41—C46	−35.6 (2)
P1—Ru1—O2—C2	−94.08 (13)	C61—P2—C41—C42	14.3 (2)
P2—Ru1—O2—C2	161.18 (13)	C51—P2—C41—C42	−88.3 (2)
Ru1—O2—C2—N1	−7.1 (2)	Ru1—P2—C41—C42	144.35 (19)
Ru1—O2—C2—C3	172.56 (18)	C46—C41—C42—C43	1.1 (4)
C6—N1—C2—O2	−8.6 (3)	P2—C41—C42—C43	−178.9 (2)
C5—N1—C2—O2	178.5 (2)	C41—C42—C43—C44	−0.8 (4)
C6—N1—C2—C3	171.72 (18)	C42—C43—C44—C45	0.1 (4)
C5—N1—C2—C3	−1.1 (3)	C43—C44—C45—C46	0.3 (4)
O2—C2—C3—C4	−162.1 (2)	C44—C45—C46—C41	0.0 (4)
N1—C2—C3—C4	17.6 (2)	C42—C41—C46—C45	−0.7 (4)
C2—C3—C4—C5	−26.3 (2)	P2—C41—C46—C45	179.3 (2)
C2—N1—C5—C4	−16.1 (3)	C61—P2—C51—C52	105.11 (18)
C6—N1—C5—C4	171.8 (2)	C41—P2—C51—C52	−148.73 (18)
C3—C4—C5—N1	25.8 (2)	Ru1—P2—C51—C52	−20.5 (2)
C2—N1—C6—C7	−57.9 (3)	C61—P2—C51—C56	−70.44 (19)
C5—N1—C6—C7	113.8 (2)	C41—P2—C51—C56	35.7 (2)
C2—N1—C6—Ru1	19.4 (2)	Ru1—P2—C51—C56	164.00 (15)
C5—N1—C6—Ru1	−168.89 (18)	C56—C51—C52—C53	2.1 (3)
C1—Ru1—C6—N1	158.30 (15)	P2—C51—C52—C53	−173.67 (19)
C7—Ru1—C6—N1	−112.4 (2)	C51—C52—C53—C54	−1.7 (4)
O2—Ru1—C6—N1	−16.09 (13)	C52—C53—C54—C55	0.1 (4)
P1—Ru1—C6—N1	64.56 (15)	C53—C54—C55—C56	1.0 (3)
P2—Ru1—C6—N1	−99.09 (16)	C54—C55—C56—C51	−0.7 (3)
C1—Ru1—C6—C7	−89.28 (13)	C52—C51—C56—C55	−0.9 (3)
O2—Ru1—C6—C7	96.33 (13)	P2—C51—C56—C55	174.67 (17)
P1—Ru1—C6—C7	176.98 (11)	C41—P2—C61—C62	−135.17 (17)
P2—Ru1—C6—C7	13.33 (19)	C51—P2—C61—C62	−30.84 (18)
N1—C6—C7—Ru1	100.72 (16)	Ru1—P2—C61—C62	94.29 (17)
C1—Ru1—C7—C6	93.33 (13)	C41—P2—C61—C66	54.18 (18)
O2—Ru1—C7—C6	−76.93 (12)	C51—P2—C61—C66	158.51 (16)
P1—Ru1—C7—C6	−5.09 (18)	Ru1—P2—C61—C66	−76.36 (17)
P2—Ru1—C7—C6	−172.55 (11)	C66—C61—C62—C63	1.0 (3)
C31—P1—C11—C12	109.99 (19)	P2—C61—C62—C63	−169.81 (16)
C21—P1—C11—C12	5.2 (2)	C61—C62—C63—C64	−0.2 (3)
Ru1—P1—C11—C12	−122.56 (17)	C62—C63—C64—C65	−0.5 (3)
C31—P1—C11—C16	−69.70 (18)	C63—C64—C65—C66	0.5 (3)
C21—P1—C11—C16	−174.53 (17)	C64—C65—C66—C61	0.3 (3)
Ru1—P1—C11—C16	57.76 (18)	C62—C61—C66—C65	−1.0 (3)
C16—C11—C12—C13	−0.4 (3)	P2—C61—C66—C65	169.86 (16)

### Hydrogen-bond geometry (Å, º)

Cg1, Cg2 and Cg3 are the centroids of the C21–C26, C41–C46 and C61–C66 rings, respectively.

**Table:**

*D*—H···*A*	*D*—H	H···*A*	*D*···*A*	*D*—H···*A*
C36—H36*A*···*Cg*3	0.95	2.68	3.490 (2)	144
C66—H66*A*···*Cg*1	0.95	2.61	3.384 (3)	139
C4—H4*B*···*Cg*2^i^	0.99	2.67	3.585 (3)	154
C14—H14*A*···*Cg*3^ii^	0.95	2.90	3.693 (3)	142

Symmetry codes: (i) −*x*+2, −*y*, −*z*+1; (ii) −*x*, −*y*−1, −*z*.
